# Thyroid follicular adenomas and carcinomas: molecular profiling provides evidence for a continuous evolution

**DOI:** 10.18632/oncotarget.23130

**Published:** 2017-12-08

**Authors:** Geneviève Dom, Sandra Frank, Sebastien Floor, Pashalina Kehagias, Frederick Libert, Catherine Hoang, Guy Andry, Alex Spinette, Ligia Craciun, Nicolas de Saint Aubin, Christophe Tresallet, Frederique Tissier, Frederique Savagner, Samira Majjaj, Ilse Gutierrez-Roelens, Etienne Marbaix, Jacques E. Dumont, Carine Maenhaut

**Affiliations:** ^1^ Institute of Interdisciplinary Research (IRIBHM), Université libre de Bruxelles (ULB), Brussels, Belgium; ^2^ WELBIO, School of Medicine, Université libre de Bruxelles, Brussels, Belgium; ^3^ Hôpital Pitié-Salpêtrière, Université Pierre et Marie Curie, Paris, France; ^4^ Institut Jules Bordet, Brussels, Belgium; ^5^ IFB, Hôpital Toulouse Purpan, Toulouse, France; ^6^ Biolibrary of the King Albert II Institute, Cliniques Universitaires Saint-Luc, and Institut de Duve, Université Catholique de Louvain, Brussels, Belgium

**Keywords:** thyroid follicular carcinoma, thyroid follicular adenoma, malignant progression mRNA, miRNA

## Abstract

Non-autonomous thyroid nodules are common in the general population with a proportion found to be cancerous. A current challenge in the field is to be able to distinguish benign adenoma (FA) from preoperatively malignant thyroid follicular carcinoma (FTC), which are very similar both histologically and genetically. One controversial issue, which is currently not understood, is whether both tumor types represent different molecular entities or rather a biological continuum.

To gain a better insight into FA and FTC tumorigenesis, we defined their molecular profiles by mRNA and miRNA microarray. Expression data were analyzed, validated by qRT-PCR and compared with previously published data sets.

The majority of deregulated mRNAs were common between FA and FTC and were downregulated, however FTC showed additional deregulated mRNA. Both types of tumors share deregulated pathways, molecular functions and biological processes. The additional deregulations in FTC include the lipid transport process that may be involved in tumor progression. The strongest candidate genes which may be able to discriminate follicular adenomas and carcinomas, CRABP1, FABP4 and HMGA2, were validated in independent samples by qRT-PCR and immunohistochemistry. However, they were not able to adequately classify FA or FTC, supporting the notion of continuous evolving tumors, whereby FA and FTC appear to show quantitative rather than qualitative changes. Conversely, miRNA expression profiles showed few dysregulations in FTC, and even fewer in FA, suggesting that miRNA play a minor, if any, role in tumor progression.

## INTRODUCTION

Thyroid nodules are very common in the general population (affecting up to 40% of people over 60 years old). Among them, follicular adenoma (FA) and follicular carcinomas (FTC) present a particular diagnostic challenge with many cases designated as being indeterminate or suspicious [1]. FA and FTC are both follicular differentiated thyroid tumors, but FA are benign tumors whereas FTC are malignant, able to metastasize via the blood stream, and can evolve into dedifferentiated aggressive tumors. FTC cannot be discriminated from the benign FA on the basis of architectural or cellular criteria. Indeed, for these follicular patterned lesions, the distinction between benign (FA) and malignant (FTC) tumors is based on the presence of vascular and/or capsular invasion [2] which demonstrates the invasive characteristics of the tumor, and will require surgery. Currently both FA and FTC are described as two distinct tumoral entities [3, 4], while other studies suggest a continuum leading from adenoma to carcinoma [5]. Since a long time, attempts have been made to discriminate FA from FTC, or more generally benign from malignant nodules, especially using fine needle aspiration (FNA) from tissue samples, however, indeterminate and suspicious cases persist. In fact, molecular characterization based on mutational profiles still leaves at least 14% of follicular neoplasms that cannot be classified [6] and an 8% cancer risk in nodules with an indeterminate cytological diagnosis displaying no known molecular alterations [7].

Indeed, the genetic alterations that are most often encountered are present in both FA and FTC, namely RAS mutations—present in 20–40 % of FA and 40–50 % of FTC- and PAX8-PPARγ rearrangements—reported in 10% of FA and 30–40% of FTC [8]. This sharing of genetic alterations combined with similar cytological and architectural features in FA and FTC account for inconclusive diagnoses for a fraction of the FNA samples, and a definitive indication on the benign or malignant character of these follicular tumors is not possible. In the recently proposed ThyroSeq NGS panel, that was designed to target 284 mutational hotspots in 12 cancer genes, 6% of the benign nodules—including FA—were positive for the detection of point mutations [9]. Given the fact that genetic variants are found in both benign and malignant nodules [10], current research remains focussed on the use of RNA-based expression classifiers, to identify suspicious nodules. Some expression based classifiers exist but lack sensitivity and/or specificity [11] (for example, three diagnostic tests were recently developed in the US, based on molecular signatures that aim to distinguish benign and malignant nodules). So far, all the mRNA based classifiers, namely Affirma (Veracyte) Alexander et al. [12], or miRNA based classifiers such as ThyGenX/ThyraMIR (Interspace Diagnostics) [13] that combine research for mutations by multiplex PCR and miRNA expression or RosettaGX Reveal™ [14] (Rosetta Genomics Ltd) and ThyroSeq (CBLPATH) [15] based only on a larger mutation panel detected by NGS, have proven to have some weaknesses.

Taking this into account, we have performed molecular profiling (mRNA and miRNA) of thyroid follicular adenoma and carcinoma, in order to further investigate the notion of continuous versus distinct evolution of these tumors. Our results support the existence of a biological continuum, in terms of RNA and miRNA deregulation, and further at the level of deregulated molecular functions and pathways. This study presents new opportunities for the investigation of oncogenic processes leading to FA progression into FTC.

## RESULTS

### Most deregulated mRNAs are common between FA and FTC

**Figure 1:**
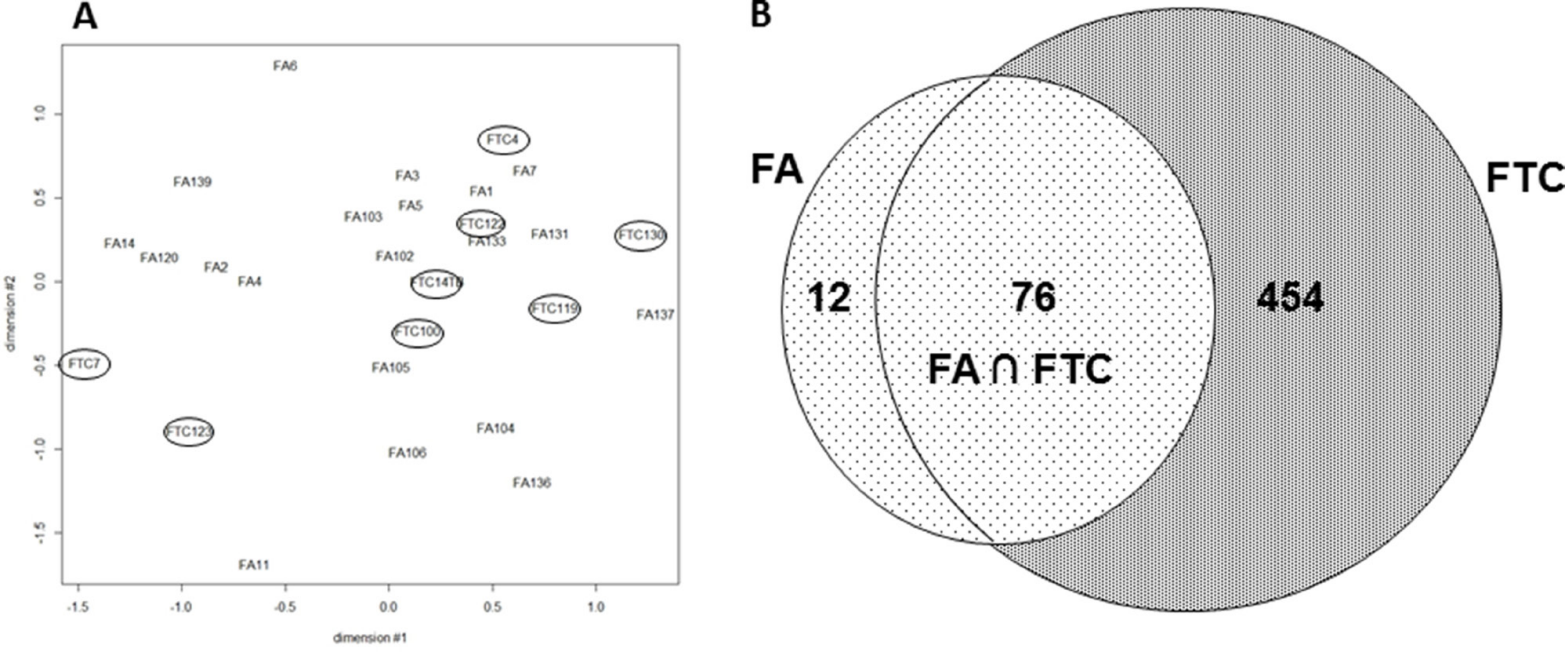
(**A**) Multidimensional Scaling (MDS) of the mRNA expression values from 20 FA and 8 FTC: all the probes present on the array were considered (FTC are encircled). (**B**) FTC and FA deregulated mRNA (fold change ≥|2| q-value ≤ 5%). Venn diagram of the significantly regulated mRNA in 20 FA and 8 FTC hybridized on Heebo slides (SAM 1 class analysis, R bioconductor).

20 FA and 8 FTC were hybridized onto double channel microarrays (HEEBO), alongside their normal adjacent thyroid tissue (clinical data, Supplementary Table 1). We first assessed the mutational status of the most common reported genetic alterations, finding few mutated samples (Supplementary Table 1). We next assessed global expression differences between FA and FTC, that is extensive differences detectable when all the genes present on the arrays were considered. An MDS analysis was performed and did not allow us to clearly distinguish both tumor types (Figure 1A). Similar results were obtained when using published FA-FTC datasets (Supplementary Figure 1). Thus FA and FTC have similar expression profiles when compared on a global scale.

To look for consistent differentially expressed genes between tumor and normal tissues, we used SAM (Significant Analysis of Microarrays) one class, a supervised classification method. Among the 88 mRNA with deregulated expression in FA (fold change ≥ |2|, *q* value ≤ 5%), 76 (86%) were also deregulated in FTC (Figure 1B). FTC, interestingly, were found overall to have a much larger number of modulated mRNA (530). Most of the deregulated mRNA were down-regulated compared to normal adjacent tissues (83% in FA, and 90% in FTC) and when modulated in the same direction in both types of tumors, which was the most frequent case, the level of deregulation for a given mRNA was higher in FTC than in FA.

Next, to compare FA and FTC mRNA expression, a SAM 2 class analysis was performed which detected 294 genes differentially expressed between both tumors (*q* value ≤ 5%) (Supplementary Table 2) with a fold change of 2 (908 with a fold change ≥ |1.5|). Again, a majority were more strongly downregulated in FTC compared with FA (94%).

**Figure 2:**
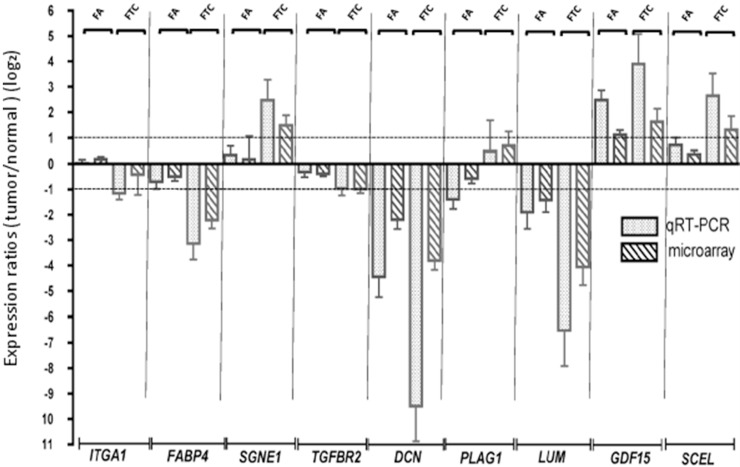
Confirmation of the microarray data by qRT-PCR Validation of the modulation of 9 genes by qRT-PCR. The microarray expressions are also represented. Log2 ratios represent the expression ratios of the genes in the tumors versus normal adjacent tissues. Error bars represent the standard deviation.

We confirmed our microarray results by qRT-PCR for 9 modulated mRNAs (Figure 2); 7 of them, FABP4, SGNE1, TGFBR2, DCN, LUM, GDF15, and SCEL were modulated in the same direction for FA and FTC but with a higher amplitude in FTC; ITGA was not modulated in FA but downregulated in FTC, and PLAG1 was slightly downregulated in FA and upregulated in FTC.

### Deregulated pathways are partially shared between FA and FTC

The genes deregulated in FA and FTC were submitted to a Pathway Analysis (David Database) [16], revealing enriched gene ontologies and pathways shared between FA and FTC (Table 1). For biological processes, categories such as cell adhesion, extracellular matrix organization and blood vessel morphogenesis were enriched in both tumor types, while some additional categories were only enriched in FTC, such as angiogenesis and cell migration. Most molecular functions and KEGG pathways were also common to both tumor types, with no deregulations specific for FA, but additional categories specific in FTC such as lipid transporter activity, focal adhesion and ECM receptor interaction.

**Table 1:** David database analysis of the deregulated mRNA in FA and in FTC

Gene Ontology Biological process	*p* val FA	*p* val FTC
GO:0007155~cell adhesion	3.97E-04	6.95E-10
GO:0007610~behavior	3.39E-04	1.84E-03
GO:0007626~locomotory behavior	1.83E-03	1.61E-03
GO:0009611~response to wounding	1.76E-04	2.61E-12
GO:0022610~biological adhesion	4.02E-04	7.13E-10
GO:0030198~extracellular matrix organization	1.30E-04	3.28E-07
GO:0043062~extracellular structure organization	1.17E-04	1.59E-05
GO:0048514~blood vessel morphogenesis	3.18E-03	1.22E-10
**GO:0001525~angiogenesis**	*0.03*	**1.91E-07**
**GO:0016477~cell migration**	*0.04*	**1.33E-07**
**Gene Ontology molecular functions**	***p* val FA**	***p* val FTC**
GO:0005125~cytokine activity	2.47E-04	7.48E-02
GO:0005539~glycosaminoglycan binding	3.73E-03	5.46E-06
GO:0008009~chemokine activity	1.18E-03	1.16E-03
GO:0008083~growth factor activity	8.04E-04	8.02E-05
GO:0042379~chemokine receptor binding	1.42E-03	1.62E-03
**GO:0005319~lipid transporter activity**	–	**5.81E-03**
**Kegg Pathway**	***p* val FA**	***p* val FTC**
hsa04060:Cytokine-cytokine receptor interaction	1.29E-02	5.26E-04
**hsa04010:MAPK signaling pathway**	–	**7.70E-05**
**hsa04510:Focal adhesion**	–	**6.69E-04**
**hsa04610:Complement and coagulation cascades**	–	**7.92E-04**
**hsa04512:ECM-receptor interaction**	*0.077296819*	**2.86E-03**

David database pathway analysis of the genes regulated with a fold change ≥ |2|, and a *q* value ≤ 5%, in FA and in FTC. Common deregulated Gene ontology categories and KEGG Patways for FA and FTC, and categories/pathways restricted to FTC (bold, underlined).

In addition, analysis of genes that were deregulated in FTC compared to normal tissues, utilizing the PANTHER database, revealed a recurrent enrichment of the Gene Ontology category LIPID TRANSPORT (GO: 0006869) with both types of microarray studies (HEEBO and Affymetrix enrichment fold 2.53 and 1.94 *p* value: 1.25E-0.3 and 2.26E-0. respectively) (data not shown).

### Our differentially expressed genes are regulated in other FA and FTC datasets, and conversely

We considered the identified differentially expressed genes between FA and FTC (fold change ≥ |2|) as a gene set and evaluated their collective expression with GSEA in the publicly available datasets of other studies from Borup et al., Finley et al., Weber et al., and Giordano et al. [17–20]. Collectively, these genes were significantly regulated in the same direction in the Finley data set (*q* value = 0.066, NES = −1.371), and the Giordano dataset (*q* value = 0.0101, NES = −1.527), and were in line (though not statistically significant) with the Borup dataset (*q* value = 0.157, NES = −1.316), and the Weber data set (*q* value = 0.327, NES = −1.170) (Supplementary Figure 2A). These results showed that our signature is not restricted to our data set but is consistent with other reported works.

Taking the inverse approach, we used the genes deregulated between FA and FTC in other datasets as genesets to evaluate their enrichment in our dataset: GSEA revealed a significant enrichment of the signatures of Borup [17] and Alexander [12] in our data (Supplementary Figure 2B).

### Despite the identification of differential gene expression, satisfying discrimination of FA and FTC could not be obtained

**Figure 3:**
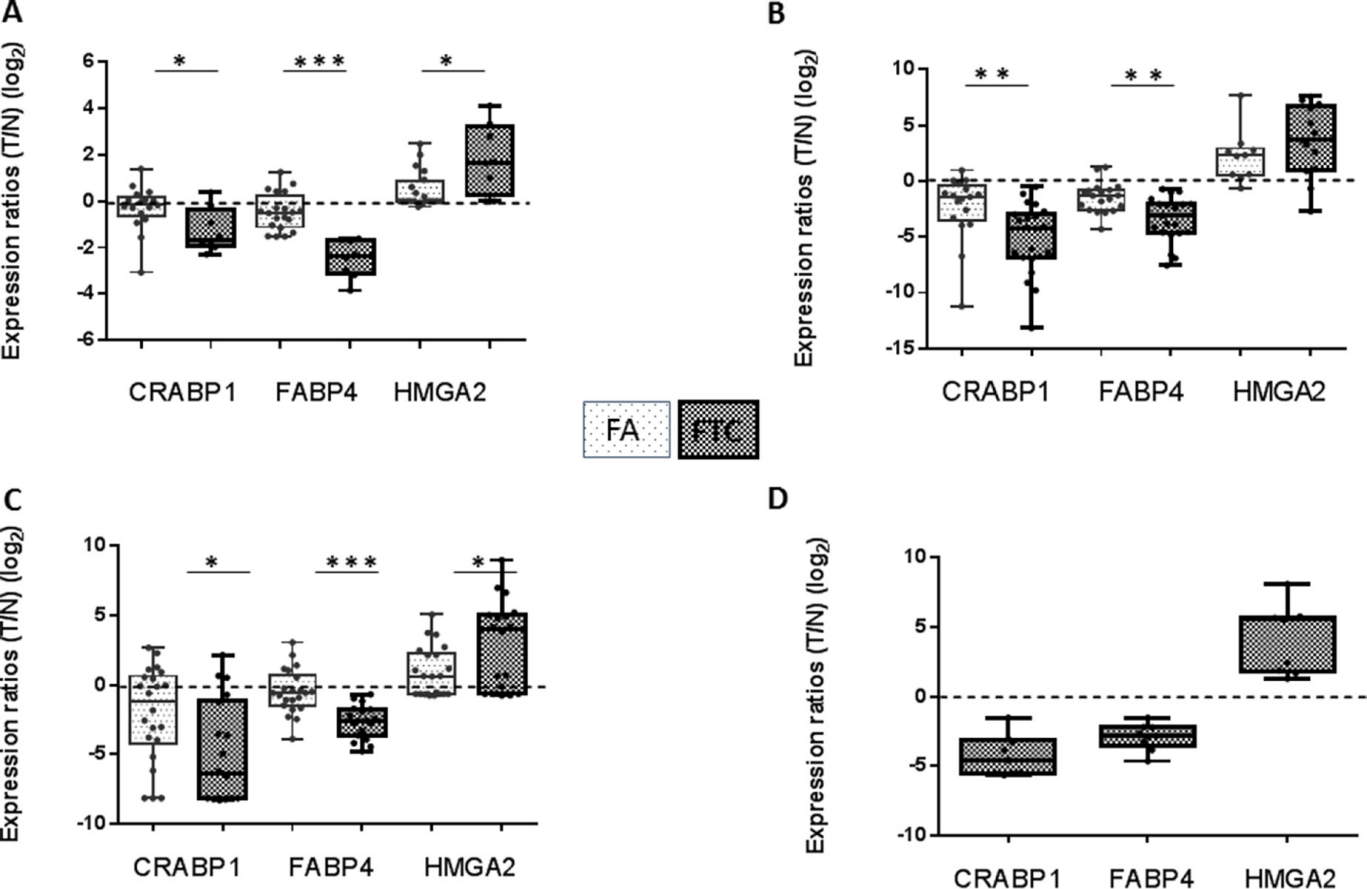
Expression ratios (tumor/normal) (log_2_) of CRABP1, FABP4, and HMGA2 in various data sets of FA and FTC (**A**) our HEEBO microarray results. (**B**) qRT-PCR on independent samples. (**C**) Borup's Affymetrix microarray data [17] (nFA = 22, nFTC = 18). (**D**) our Affymetrix microarray data (*n* = 9). (T: tumor; N: normal).

**Figure 4:**
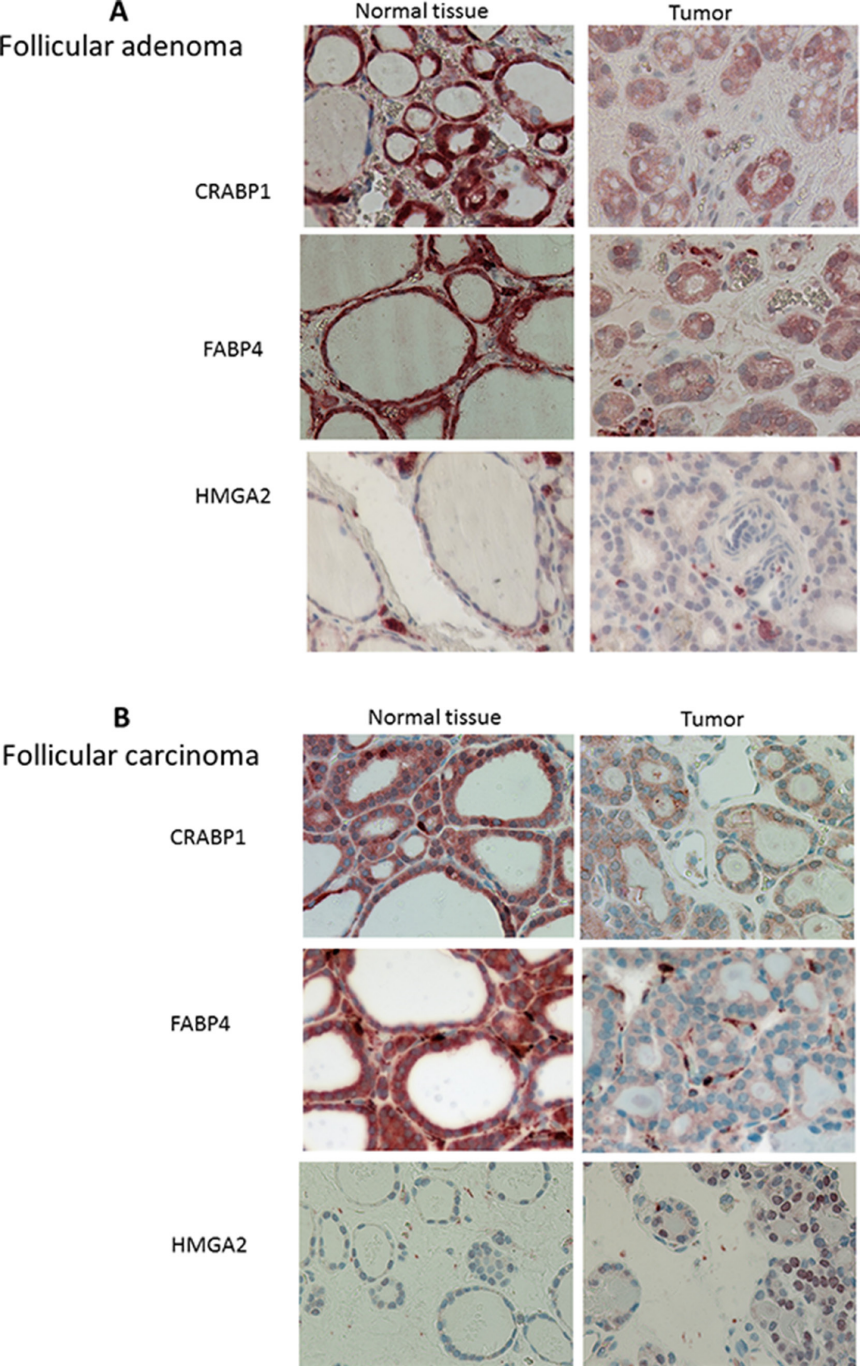
Immunolabelling of follicular adenomas (*n* = 16) (**A**) and follicular carcinoma (*n* = 17) (**B**) and normal adjacent tissues for CRABP1, FABP4 and HMGA2. Magnification 40×.

Among the genes differentially expressed between FA and FTC, we focused on three genes that were little modulated in FA, but clearly downregulated (CRABP1, FABP4) or upregulated (HMGA2) in FTC (Figure 3A). The downregulation of CRABP1 and FABP4, as well as the upregulation of HMGA2 were validated by qRT-PCR on the same samples (data not shown), using independent samples for CRABP1 and FABP4 (Figure 3B), in data from the literature [17] (Figure 3C), and in our Affymetrix data for the FTC (Figure 3D). Although most of the results were statistically significant, it was obvious that outliers were present among FA and FTC. We subsequently confirmed their modulation at the protein level by immunohistochemistry (Figure 4A and 4B): cytoplasmic staining of CRABP1 was increased in follicular adenoma, and FABP4 expression was reduced in adenoma and further reduced in carcinoma. HMGA2 was not present in normal tissues and in follicular adenoma, but was detected in the nucleus of most FTC.

To further investigate the potential of these three genes to discriminate FA from FTC, the KNNX validation algorithm was applied to our microarray data (8 FTC and 20 FA) and on the data from Borup et al. (10 FTC and 11 FA) [17]. This allowed us to correctly classify 73% of FTC and 86% of FA (Table 2). Performing a similar analysis with our qRT-PCR data (11 FTC and 8 FA) resulted in a correct classification of 91% of FTC and 88% of FA. However, these results were based on the analysis of a limited number of samples, precluding definitive conclusions (Supplementary Table 3).

**Table 2:** KNNX validation classification of FA (*n* = 42) and FTC (*n* = 26) samples

		Predicted	
		FTC	FA
True	FTC (26)	73% (19)	27% (7)
	FA (42)	14% (6)	83% (36)

The supervised classification (K-nearest neighbors classification with leave one out cross-validation) was run with the T/N expression ratios of CRABP1, FABP4, and HMGA2 derived from our microarray data and from Borup's data [17]. Confusion matrix showed that 73% of FTC and 86% of FA were well classified.

### MiRNA analysis revealed few deregulated miRNA in FTC and fewer in FA

To further analyze the molecular phenotype of follicular tumors, miRNA expression profiling was performed on 10 FA and 9 FTC. The tumor samples and their normal adjacent tissues were hybridized onto in-house printed microarrays covering 841 human miRNA. SAM analyses were used to obtain lists of deregulated miRNAs (q-val ≤ 5% and fold change ≥|1.5|) (Table 3A and 3B).

**Table 3:** list of miRNA deregulated in FA and FTC

A		B	
miRNA deregulated in FTC	Log2 Ratio	miRNA upregulated in FTC vs FA	Fold Change
hsa-miR-140-3p	0.615	hsa-miR-129-1-3p	2.847
hsa-miR-138-3p	0.989	hsa-miR-138-1-3p	2.091
hsa-miR-937-3p	1.013	hsa-miR-600	2.005
hsa-miR-129-1-3p	1.235	hsa-miR-135a-5p	2.417
hsa-miR-600	0.881	hsa-miR-125b-5p	1.976
hsa-miR-220a	1.982	hsa-miR-551b-3p	1.931
hsa-miR-129-2-3p	1.977	hsa-miR-1273a	1.618
hsa-miR-340-5p	0.724	hsa-miR-377-3p	1.590
hsa-miR-933	0.775	hsa-miR-27a-3p	1.584
hsa-miR-640	−1.063	hsa-miR-616-5p	1.591
hsa-miR-1275	−0.931	hsa-miR-23a-3p	1.451
hsa-miR-326	−1.069	hsa-miR-491-3p	1,946
hsa-miR-508-5p	−0.776	**miRNA downregulated in FTC vs FA**	**Fold Change**
hsa-miR-542-5p	−1.070	hsa-miR-542-5p	0.485
hsa-miR-154-3p	−0.692	hsa-miR-155-5p	0.444
hsa-miR-554	−0.591	hsa-miR-640	0.439
**miRNA deregulated in FA**	**Log2 Ratio**	hsa-miR-154-3p	0.474
hsa-miR-215-5p	0.645	hsa-miR-326	0.495
hsa-miR-155-5p	0.809	hsa-miR-631	0.413
hsa-miR-144-3p	−0.848	hsa-miR-1275	0.551
hsa-miR-451a	−1.049	hsa-miR-509-3-5p	0.611
		hsa-miR-508-5p	0.654

A. miRNAs significantly up (≥ 1,5 x) and down (≤ 1,5 x)-regulated (q val ≤ 5% in FTC, 20% in FA) in FTC and in FA following SAM 1 class analysis. B. miRNAs significantly up (≥ 1,5 x) and down (≤ 1,5 x) -regulated (q val ≤ 5%) in FTC versus FA following SAM2 class analysis.

**Figure 5:**
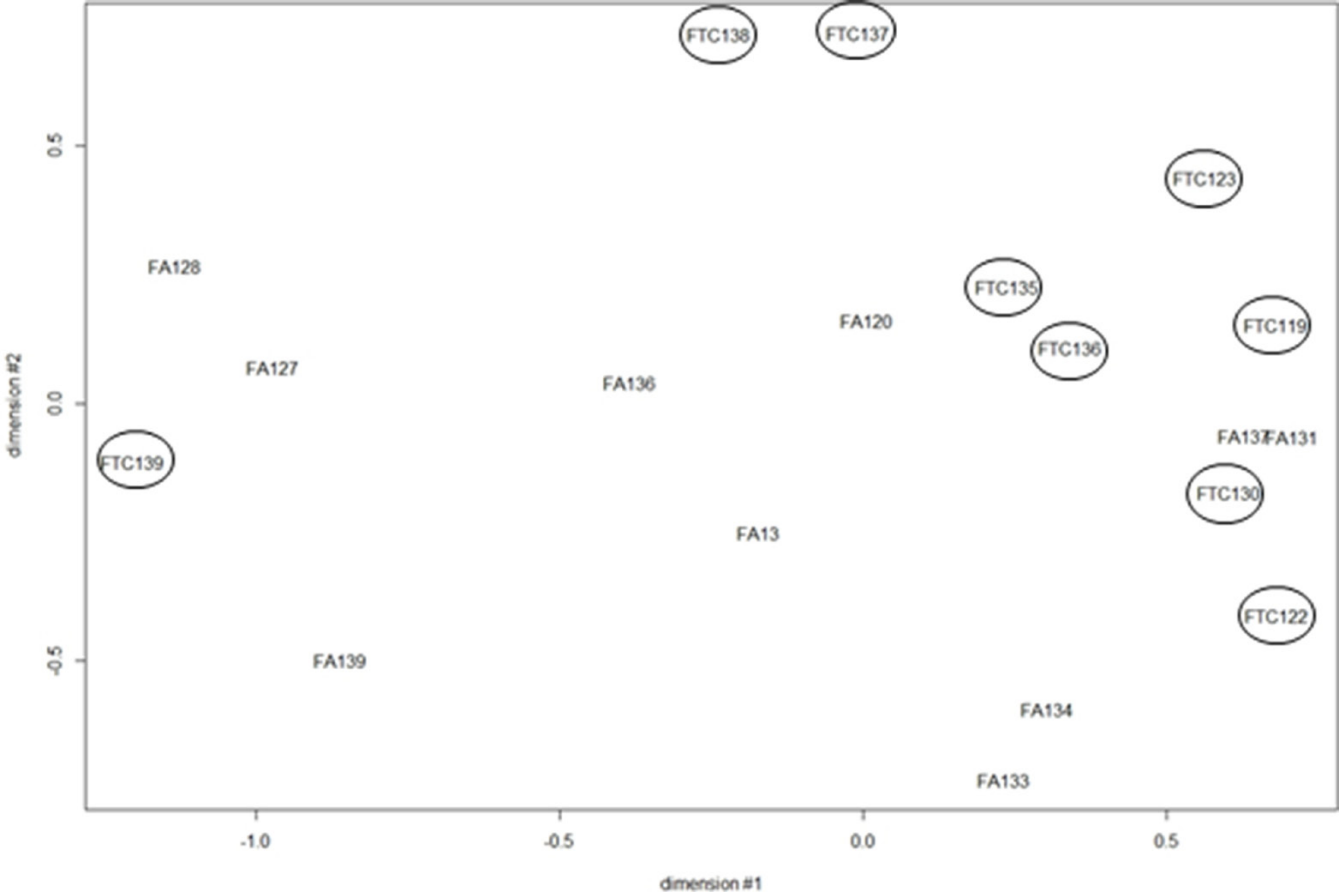
Multidimensional scaling (MDS) of the miRNA expression data from 10 FA and 9 FTC All the probes present on the array were considered (FTC are encircled).

A multidimensional scaling analysis performed with all the miRNA expression data showed a good separation between FA and FTC with the exception of 3 samples: FTC 139, FA 131, FA 137 (Figure 5). Among these, FA 131 and FA 137 mixed with the FTCs had no particular clinical features that would justify their exclusion and were kept for further analyses. On the other hand FTC 139 presented an important inflammatory reaction (thyroiditis) known to provoke large transcriptomic perturbations [21]. Nevertheless, when looking at differentially expressed miRNA in the tumors compared to their normal counterparts using SAM 1 class, only a few miRNAs were regulated consistently with a fold change ≥|1.5| and *q* value ≤ 5% across all the samples of the same class of tumors: 16 for the FTC and none for the FA (4 if a *q* value of 20% was accepted) (Table 3A). No single commonly deregulated miRNA was found.

To search for miRNA differentially expressed between FA and FTC, a SAM 2 class analysis was performed. Only 12 miRNA were upregulated in FTC compared to FA (fold change ≥|1.5| *q* value ≤ 5%), and 9 miRNA were downregulated (Table 3B), only two of them, miR-631 and miR-135a-5p were also deregulated respectively in the studies of Rossing [22] and Stokowi [23], albeit in the opposite direction. Remarkably, analysis of published data sets revealed very few commonly regulated miRNA [22–28] (Supplementary Table 4).

In a next step we investigated the negative correlation between mRNA and miRNA expressions; indeed, the biological action of miRNA is mainly carried out by silencing the expression of their target genes. Table 4 shows the intersection of the mRNA significantly deregulated between FA and FTC (our experimental data), and the targets of the miRNAs significantly deregulated between FA and FTC, obtained by miRDB tool (http://mirdb.org/miRDB/): among the mRNA targets of the 12 upregulated miRNA in FTC vs FA, 105 were present in our list of downregulated mRNA. Similarly, among the mRNA targets of the 9 downregulated miRNA, 4 were present in our list of upregulated mRNA. A David database analysis was performed to investigate the pathways altered by these 109 mRNA and revealed an enrichment in the GO category “regulation of kinase activity” (Supplementary Table 5).

**Table 4:** list of mRNA differentially regulated between FA and FTC, which are targets of miRNA differentially regulated between FA and FTC

A				B
CRIM1	KL	PTP4A2	HBEGF	DCLRE1A
EPAS1	TMEM47	GNG2	STEAP2	SLC16A9
EBF3	FGD4	GULP1	CAPZA2	ELOVL2
ANTXR1	CALCRL	RPS6KA5	CLMN	CCNG1
LPP	SEMA3D	ATP6V1E1	CLU	
BVES	QKI	SYNPO2	VLDLR	
FEZ1	ENPEP	PPP1R12B	NLN	
BCLAF1	PRKD3	ZCCHC10	EI24	
LHFP	SLC14A1	MAN1A1	PDIA5	
BAALC	BTN3A3	PCDH18	HDAC2	
CD34	SEC23A	POGZ	SHPRH	
CXCL12	PAPSS2	OSBPL9	NR3C1	
KCTD12	C1orf52	BIN2	SLC1A1	
MYCT1	PRKCA	NAP1L5	KIAA1274	
CLIC4	FMO2	CBX7	ADH5	
TSPAN8	DICER1	NFATC3	PLDN	
TNFRSF11B	STARD13	PELI1	PACSIN2	
AP1S2	RARRES1	NBN	PTX3	
JAK2	SSR1	PKIA	NR2F2	
CYP20A1	PTPRB	STIM2	NCOA1	
H3F3B	SMAD9	SSH1	ZNF626	
GAB1	ZNF37A	PDE7A	KHDRBS2	
TSPAN12	PRKX	MYO10	NFYB	
EPHA3	PAPOLG	LATS2	HNMT	
CA5B		CDH11	CLDN8	
		ITGAX	ABI3BP	
		KLF13	MLL3	
		CLYBL	PSMA1	

A: mRNA that are downregulated in FTC vs FA and targets of miRNA upregulated in FTC vs FA.

B: mRNA that are upregulated in FTC vs FA and targets of miRNA downregulated in FTC vs FA.

## DISCUSSION

The differential diagnosis between FA and FTC is currently a major clinical challenge. These two tumors are highly similar when assessing histopathology [29, 30] or immunohistochemistry [31–34], as well as gene or protein expression [35, 36]. For several years, it has been debated within the field as to whether these two tumors are distinct molecular entities or represent a biological continuum.

To address this point, we defined the molecular phenotype of FA and FTC, both at mRNA and miRNA levels, by microarray analyses. On a global scale, no qualitative distinction between the transcriptomes of FA and FTC was observed. Analyzed by MDS, the mRNA expression data from many published studies also displayed different degrees of overlap for both tumor types. This contrasts with papillary or anaplastic carcinomas, which show specific gene modulations as well as many genes deregulated in opposite directions [37]. However, when using a supervised approach (SAM), differentially expressed genes between FA and FTC were detected.

Our data did not allow us to identify a molecular signature discriminating FA from FTC satisfactorily but, on the contrary, suggest that most FTC derive from FA, and that deregulation of expression in the tissue is gradual from benign to malignant tumors (as already suggested by others) [38, 39]. This is reflected in our microarray data which shows a large proportion of common deregulations between FA and FTC. Furthermore, most of the mRNA differentially regulated between FA and FTC were modulated in the same direction, with the extent of modulation stronger in FTC. In addition, both types of tumor share the same perturbations of signaling pathways. Of course, FTC, as more advanced tumors, display additional dysregulated genes and pathways. One of the most striking differences between FA and FTC is invasion and migration; this is reflected in our gene expression data, where genes associated with cell migration, focal adhesion, and ECM-receptor interaction are specifically highlighted in the carcinomas.

We focused on 3 differentially expressed genes which could have been potentially able to discriminate FA from FTC, on the basis of different criteria such as the extent of modulation and information found in the literature concerning their potential implication in cancers. HMGA2 or High Mobility Group AT-Hook 2 is a nuclear non histone chromatin-associated protein that acts as a transcription factor regulating the expression of various genes; this gene is not expressed in normal tissues, but is expressed in malignant tumors and has been proposed as a malignancy marker in thyroid [33, 40–44] and other types of cancers [45, 46]. Its potential oncogenic role has been noted in previous studies [46–48]. CRABP1 or Cellular Retinoic Acid Binding Protein 1, is a protein that allows the transport of retinoic acid from the cytoplasm to the nucleus and has an important role in development. We have previously shown that this gene is part of a thyrocyte differentiation index [49]. As for the CRABP1 paralog FABP4, its expression in thyroid tumors seems to be inversely correlated to the malignancy or aggressiveness of the tumors and has been used in general classifiers proposed to discriminate malignant from benign thyroid tissues [21, 50]. FABP4 (Fatty Acid Binding Protein 4) is a cytoplasmic protein, that binds long chain fatty acids and, as with CRABP1, FABP4 also binds retinoic acid. It plays a role in lipid transport and metabolism in adipocytes, and is associated with insulin resistance, type 2 diabetes and cardiovascular disease [51]. It is predominantly expressed in mature adipocytes and in macrophages, and its expression is induced by the transcription factor PPARγ [52]. This protein is also described as a partner of PTEN [53] and is downregulated in PTC [54].

The combination of HMGA2, CRABP1, and FABP4 expression did not allow us to completely separate the independent FA and FTC samples. Furthermore, immunohistochemistry experiments performed for the corresponding proteins showed for some of the samples atypical expression, suggesting that these could be intermediate between FA and FTC (data not shown). This low selectivity is also reflected by the qRT-PCR results with, for instance, some adenomas overexpressing HMGA2.

As FABP4 is a protein involved in the transport of fatty acids, the differential regulation of FABP4 in our data and also in many other studies suggests differences in lipid metabolism between FTC, FA and normal thyroid tissue. A pathway analysis with the FTC deregulated genes confirmed this modification in lipid metabolism, which might contribute to tumor progression [55, 56]. Analyses of patient sera have shown that the characteristics of the serum lipidome were different for different thyroid tumors [57]. Another study performed on FFPE samples demonstrates that lipid metabolites are globally downregulated in thyroid tumors while lactic acid production is increased, and that some fatty acids esters such as lauric acid propyl ester or other lipid metabolites such as myo-inositol phosphate could be used to distinguish FA from FTC [58]. Furthermore, aberrant lipid metabolism was recently described in anaplastic thyroid carcinoma, part of which may derive from FTC [59]. On the other hand, the PI3K/AKT signaling pathway is deregulated in 55% of FTC [60], and increased phosphorylation of AKT is generally observed [61]. PI3K/AKT is probably the main pathway involved in FTC tumorigenesis while, in papillary thyroid carcinomas, constitutive activation of the MAPK signaling pathway plays a major role. Lipid metabolism and PI3K are linked [62], and thus both are likely involved in FTC development and progression.

With regard to miRNA expression, the reduced number of commonly deregulated miRNAs in FA and FTC suggests that there is a great variability among tumors of each type and that miRNAs are not critically involved in their respective tumorigenesis. This is reflected in the MDS where tumors are quite disparate even when belonging to the same class. Concerning the miRNA differentially regulated between FA and FTC, only a few deregulations were common across different studies, including ours. For instance, the two miRNAs that were used to classify FA and FTC by the group of Stokowy, miR-7-2-3p, and miR-7-5p [23], were not differentially regulated between adenomas and carcinomas in our work (Supplementary Table 4): this variation between studies, and the small number of miRNA deregulated between the two tumor types could reflect the fact that miRNA are not key regulators in the supposed progression from FA to FTC. Accordingly, a recent review from our group showed that deregulated miRNA are far less numerous in FTC than in PTC and very few are common between different studies [63]. The small effects of normal follicular cell transformation to follicular adenoma and carcinoma in terms of miRNA deregulation is consistent with relatively moderate dedifferentiation of these tumors. It is also in agreement with our previous work showing that dedifferentiation of normal human thyrocytes in primary culture, by treatment with EGF/serum, does not greatly modify the miRNA expression profiles [64].

The concept of a biological continuum in tumor progression starting from FA evolving to FTC is supported by two strong arguments: firstly, the common predominant mutations encountered in FA and FTC, i.e. RAS mutations and PAX8-PPARγ rearrangements. Although mutations in PI3K catalytic subunit seem to be restricted to FTC (8%) [60], this gene was reported to be amplified in 12% of benign thyroid adenomas and 24% of follicular carcinomas [60]. Secondly, the transcriptomic mRNA profiles of both tumors show a large proportion of commonly deregulated genes whose deregulation is further amplified in the carcinomas. Most of the mRNA differentially regulated between FA and FTC are actually showing regulation in the same direction, or are not deregulated at all in FA.

The hypothesis of a biological continuum, i.e. adenomas and carcinomas are the same tumors but at different stages of progression, is in line with the fact that the attributed diagnosis for a particular tumor can be for one pathologist FA (suspected for later malignancy), and for another mild FTC with low malignancy score (if no invasion of the capsule is seen). Even among trained pathologists of the same laboratory, diagnoses of the same samples are sometimes different from one expert to the next [29]. So, the two classes are not definitively distinct and there is histological overlap between them, which is reflected by our different results, with cases matched perfectly with this classification but also cases which were more atypical and could be intermediates between FA and FTC, or even FVPTC. Of course these conclusions are based on data obtained with analyses on bulk tumoral material, as in most published data, a limitation that will only be overcome by the use of *in situ* methods.

Although we have conducted this study on a relatively small number of tumors, these were very carefully chosen (some were discarded after re-evaluation by pathologists). Equally we would suggest that in published studies there are likely some tumors for which diagnosis was challenging. FTC is becoming increasingly rare in the population probably due to the the disappearance of one etiologic recognized factor for FTC: iodine deficiency [65] which is now largely eradicated in many countries. However, this low number may also reflect the fact that FVPTC is currently the most frequent diagnosis even for a tumor which could have been considered as an FTC [66]: as pointed out by some authors, there may be a tendency to over-diagnose the follicular variant of PTC (FVPTC) which, as a corollary, leads to under-diagnosis of FTC (this being linked to the interpretation of the nuclear features of the tumors) (1). However, some authors have reported that the molecular profiles of FVPTC are closer to those of the FA/FTC group than to those of the classical forms of PTC [67]. This is also reflected in the TCGA data where follicular-patterned PTC (i.e. Ras-like PTCs) are different in their molecular profiles from classical PTCs (BRAF-like PTCs) leading the authors to propose a revision of the classification [68]. For others, there is a tendency to lower the threshold for the diagnosis of FVPTC, and some tumors that could be classified as FA may now be classified as FVPTC [69]. An MDS performed with the expression data of FVPTC, FA and FTC tumors of the Finley et al. study [18] displays an intermediate expression profile for FVPTC positioned among the FA and the FTC groups. When PTC expression data from this study is added to the MDS analysis, the FVPTC are positioned between the FA and the PTC group, and many of them are mixed with the FA samples (Supplementary Figure 3). Moreover, a nomenclature revision has recently been proposed for the encapsulated follicular variant of papillary thyroid cancer EFVPTC [70] which is now designated as non-invasive follicular tumor with papillary-like nuclear features (NIFTP), characterized by an indolent behavior and a very low risk of adverse outcome.

**Figure 6:**
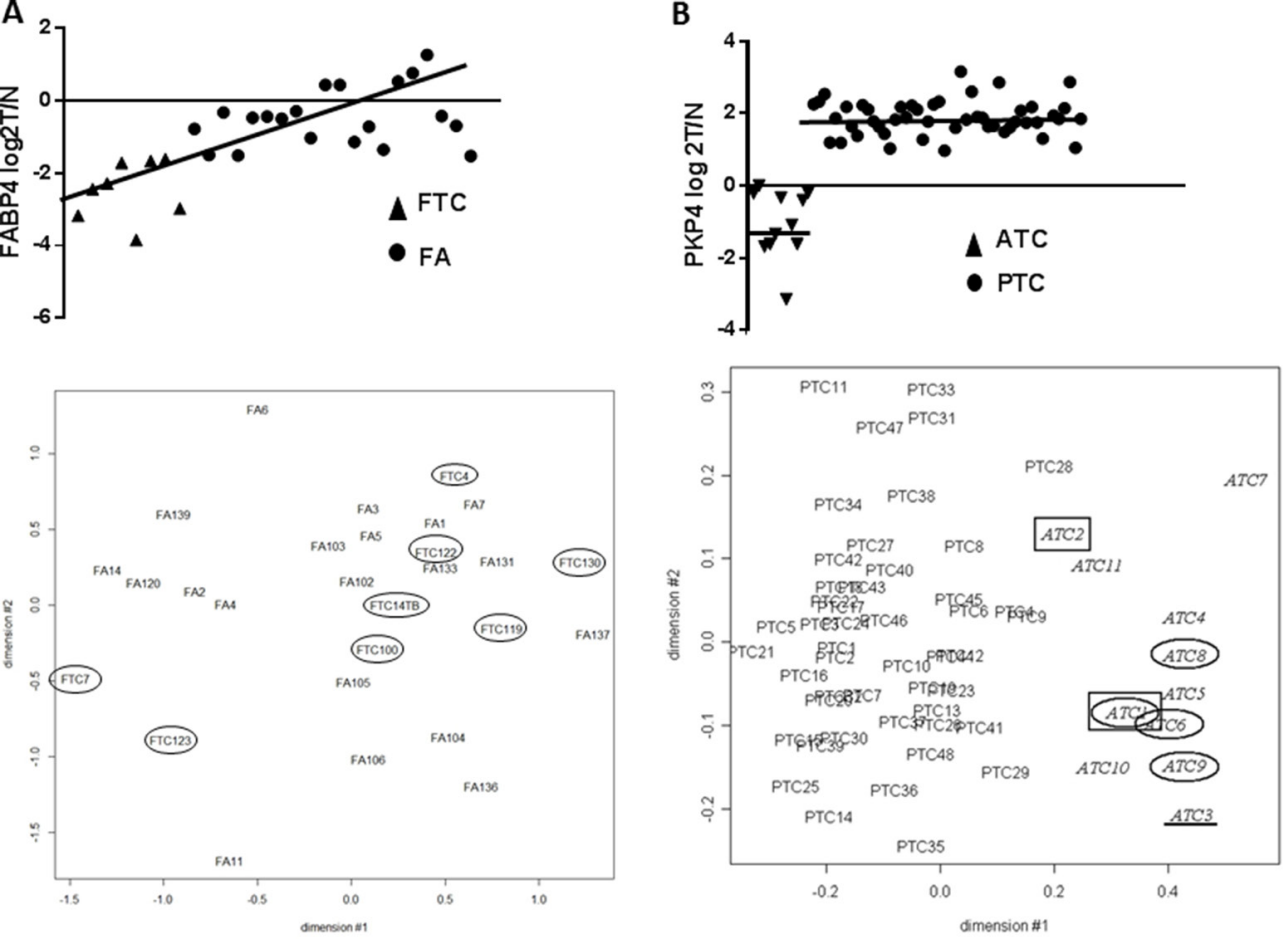
(**A**) mRNA expression of FABP4 in FA and FTC samples, and MDS with all the microarray expression data in FA and FTC samples: both one of the most performant markers and the expression data at global level highlight the idea of a continuum. (**B**) mRNA expression of PKP4 in ATC and PTC samples, and MDS with all the microarray expression data in ATC and PTC samples (37): in ATC and PTC, molecular markers split up the 2 samples groups.

In conclusion, in FA and FTC, mRNA expression as well as immunohistochemical data generally largely overlap, suggesting a biological continuum rather than a sharp transition in both types of tumors (Figure 6). This contrasts with other thyroid tumors, such as PTC and ATC, whose mRNA expression profiles showed net differences [37] although a fraction of anaplastic thyroid carcinoma can arise from papillary tumors. This progressive evolution from follicular adenoma to carcinoma could be explained by the appearance of successive multiple minor genetic or post-genetic alterations rather than a few major driver mutations [71]. Within this context, the distinction between follicular benign and malignant tumors should be based primarily on histology rather than on a molecular signature.

## MATERIALS AND METHODS

### Samples

20 follicular non-autonomous adenomas and 12 follicular carcinomas (minimally and widely invasive) were obtained from multiple clinics: Pitié-Salpêtrière (Paris, France), Jules Bordet Institute (Brussels, Belgium), CHU Angers (Angers France), Hôpital de Jolimont (La Louvière, Belgium). Diagnoses were confirmed by thorough microscopic examination of the surgical samples by the respective pathologists of the different Institutions. Tissues were immediately dissected, placed on ice, snap-frozen in liquid nitrogen and stored at −80°C until RNA processing. Protocols have been approved by the ethics committees of the Institutions. The available patient information, clinical and gene alteration data relative to these samples are presented in Supplementary Table 1. Some samples have been analysed both for mRNA and miRNA profiles, and some FTC were analysed both on HEEBO (dual channel) slides and Affymetrix.

Immunohistochemistry experiments were performed on independent samples (16 FA and 17 FTC) obtained from the Biothèque de l'Institut Roi Albert II Cliniques Universitaires Saint-Luc (Brussels, Belgium), and the Jules Bordet Institute (Brussels, Belgium).

### External data

We used the publicly available data of Borup et al [17], Giordano et al. [20], Finley et al. [18], Alexander et al. [12] and Weber et al. [19].

### Mutation screening

For most samples we defined the mutational status for known thyroid oncogenes and tumor suppressor genes [72, 73]. FA and FTC were tested for the presence of point mutations in *BRAF, NRAS, HRAS, KRAS, TP53, PI3KCA, PTEN* genes, and for the presence of PAX8/PPARγ rearrangements. The primers were designed to target the point mutations and the rearrangements most usually found in follicular thyroid carcinomas as in [74].

### RNA purification

To obtain mRNA, total RNA was extracted from thyroid tissues using Trizol reagent kit (Invitrogen), followed by purification on RNeasy columns (Qiagen). For microRNA analyses, the purification was performed using the miRNeasy minikit (Qiagen). RNA concentrations were determined by spectrophotometry, quantified, and their integrity verified using an automated gel electrophoresis system (Experion, Bio-Rad).

### mRNA microarrays

#### Double channels

After RNA amplification (using Amino Allyl MessageAmp II aRNA amplification kit Ambion, Austin, TX, USA), 8 μg of aRNA was labeled, fragmented, and hybridized for 16–18 h onto human exonic evidence-based 70-mer oligonucleotide (HEEBO) microarrays [75]. The microarray slides were washed under stringent conditions and scanned using a GenePix 4000B scanner (Axon, Sunnyvale, CA, USA). All hybridizations were performed in duplicate with dye swap with the normal adjacent tissue for the 20 FA and 8 FTC analyzed.

### Affymetrix

RNA amplification, cDNA synthesis and labelling were performed following Affymetrix (Santa Clara, CA, USA) protocol: 100 ng of RNA from 9 follicular carcinomas and a reference pool of 23 normal, non-neoplastic thyroid tissues from the contralateral lobe with respect to different thyroid tumors were hybridized on Affymetrix Human Genome U133 Plus 2.0 Arrays.

### Quantitative RT-PCR

After DNAse treatment using DNAse I amplification Grade (Invitrogen), reverse transcription was performed using Superscript II RNase H Reverse Transcriptase (Invitrogen) following the manufacturer's protocol. Primer sequences (designed with the Primer-3 software http://frodo.wi.mit.edu/primer3) are available in Supplementary Table 6. The qRT-PCR products were run on an Applied Biosystems 7500 Fast Real Time PCR with SyberGreen (Applied Biosystems). NEDD8 and TTC1 mRNA expression were used for normalization [76].

### miRNA microarrays

1μg of total RNA from 10 FA and 9 FTC was engaged for the hybridizations. Briefly, total RNA was labelled using the miRCURY LNA microRNA Power Labelling Kit (Hy3/Hy5) (Exiqon, Copenhagen, Denmark), according to the manufacturer's protocol. Labeled RNA was purified on a miRNeasy column (Qiagen), and samples were hybridized using Corning Pronto! Microarray Hybridization Kit onto in-house-printed slides with the mercury LNA microRNA ready-to-spot probes set (V11.0 according to Mirbase 11.0) from Exiqon. After overnight hybridization, microarray slides were washed under stringent conditions: twice 60s at 60°C with 2X SSC and 2% SDS, twice 60s at 60°C with 2X SSC and twice 60s at RT with 0.2X SSC. Slides were then scanned using a GenePix 4000B scanner (Axon, Sunnyvale, CA, USA). All hybridizations were performed in duplicate with dyes swapped.

### Data acquisition and bioinformatic analyses

Double channel slides were scanned using a Molecular Devices 4000B laser scanner and expression levels were quantified using GenePix Pro 6.1 image analysis software (Axon Instruments, CA, USA). For mRNA hybridization, data analyses were performed using BRB-ArrayTools [77]. Data were imported using the GenePix data importer, ratios were flipped for reverse-fluor experiments, and background adjustment was applied. Both red and green spots with intensities below the minimum and flagged spots were excluded from the analysis. Data were normalized using lowesssmoother (locally weighted scatterplot smoothing). For miRNA hybridizations, R tools were used as described previously [64]. Regulated genes were selected using significant analysis of microarrays (SAM) [78], and data were visualized using the R software version 2.11.1 [79] or GenePattern (http://www.broad.mit.edu/cancer/software/genepattern/). This second algorithm was also used for normalization of Affymetrix data (CELL files GCRMA) and other applications such as class prediction based on leave-one-out cross-validation that was performed with the k-nearest neighbours algorithm (KNNXValidation) [80]. Gene Set Enrichment Analysis (GSEA; MsigDB) was used to search for multigene signatures allowing to distinguish classes [81]. Biological significance of regulations were conducted with the DAVID (Database for Annotation, Visualisation and Integrated Discover) software [16] and PANTHER database http://pantherdb.org [82].

### Immunohistochemistry

Formaldehyde 4 % -fixed paraffin-embedded 5-μm-thick sections were deparaffinized and rehydrated. Antigen unmasking was performed in preheated 0.01 M citrate buffer (pH 6.0), 2 × 5 min inside microwave (720 W) followed by endogenous peroxidase activity blocking with 3% hydrogen peroxide in methanol. The sections were permeabilized by 15 min incubation in 0.3% Triton X-100 PBS, followed by 60 min incubation in 0.3% Triton X-100 PBS containing 3% BSA and 10% normal goat serum to block non-specific binding. Primary antibody incubation (Abcam, Cambridge, UK: HMGA2 ab52039, diluted 1/100, CRABP1 ab2816 and FABP4 ab13979 diluted 1/200) was performed overnight in a cold room. After washing, slides were incubated with Dako EnVision + System™ HRP for 60 minutes and the peroxidase reaction visualized using AEC (Dako). The sections were counterstained with hematoxylin. Omission of the primary antibody served as a negative control: sections were incubated with 0.3% Triton X-100 PBS containing 3% BSA and 10% normal goat serum, and no staining was observed. The adjacent normal thyroid tissue was used as an internal control for immunolabelling, and positive controls were also used when available: PTC (papillary thyroid cancer) for HMGA2, and white adipose tissue for FABP4. Images were obtained on an Axioimager Z1 Zeiss microscope with the 40× objective.

## SUPPLEMENTARY MATERIALS FIGURES AND TABLES









## References

[1] Sobrinho-Simões M, Eloy C, Magalhães J, Lobo C, Amaro T (2011). Follicular thyroid carcinoma. Mod Pathol.

[2] McHenry CR, Phitayakorn R (2011). Follicular adenoma and carcinoma of the thyroid gland. Oncologist.

[3] Krause K, Prawitt S, Eszlinger M, Ihling C, Sinz A, Schierle K, Gimm O, Dralle H, Steinert F, Sheu SY, Schmid KW, Fuhrer D (2011). Dissecting molecular events in thyroid neoplasia provides evidence for distinct evolution of follicular thyroid adenoma and carcinoma. Am J Pathol.

[4] Karger S, Krause K, Engelhardt C, Weidinger C, Gimm O, Dralle H, Sheu-Grabellus SY, Schmid KW, Fuhrer D (2012). Distinct pattern of oxidative DNA damage and DNA repair in follicular thyroid tumours. J Mol Endocrinol.

[5] Gupta N, Dasyam AK, Carty SE, Nikiforova MN, Ohori NP, Armstrong M, Yip L, LeBeau SO, McCoy KL, Coyne C, Stang MT, Johnson J, Ferris RL (2013). RAS mutations in thyroid FNA specimens are highly predictive of predominantly low-risk follicular-pattern cancers. J Clin Endocrinol Metab.

[6] Nikiforov YE, Ohori NP, Hodak SP, Carty SE, LeBeau SO, Ferris RL, Yip L, Seethala RR, Tublin ME, Stang MT, Coyne C, Johnson JT, Stewart AF, Nikiforova MN (2011). Impact of mutational testing on the diagnosis and management of patients with cytologically indeterminate thyroid nodules: a prospective analysis of 1056 FNA samples. J Clin Endocrinol Metab.

[7] Le Mercier M, D'Haene N, De Nève N, Blanchard O, Degand C, Rorive S, Salmon I (2015). Next-generation sequencing improves the diagnosis of thyroid FNA specimens with indeterminate cytology. Histopathology.

[8] Hsiao SJ, Nikiforov YE (2014). Molecular approaches to thyroid cancer diagnosis. Endocr Relat Cancer.

[9] Nikiforova MN, Wald AI, Roy S, Durso MB, Nikiforov YE (2013). Targeted next-generation sequencing panel (ThyroSeq) for detection of mutations in thyroid cancer. J Clin Endocrinol Metab.

[10] Pagan M, Kloos RT, Lin CF, Travers KJ, Matsuzaki H, Tom EY, Kim SY, Wong MG, Stewart AC, Huang J, Walsh PS, Monroe RJ, Kennedy GC (2016). The diagnostic application of RNA sequencing in patients with thyroid cancer: an analysis of 851 variants and 133 fusions in 524 genes. BMC Bioinformatics.

[11] Nishino M Molecular cytopathology for thyroid nodules: A review of methodology and test performance. Cancer Cytopathol.

[12] Alexander EK, Kennedy GC, Baloch ZW, Cibas ES, Chudova D, Diggans J, Friedman L, Kloos RT, LiVolsi VA, Mandel SJ, Raab SS, Rosai J, Steward DL (2012). Preoperative diagnosis of benign thyroid nodules with indeterminate cytology. N Engl J Med.

[13] Labourier E, Shifrin A, Busseniers AE, Lupo MA, Manganelli ML, Andruss B, Wylie D, Beaudenon-Huibregtse S (2015). Molecular testing for miRNA, mRNA, and DNA on fine-needle aspiration improves the preoperative diagnosis of thyroid nodules with indeterminate cytology. J Clin Endocrinol Metab.

[14] Benjamin H, Schnitzer-Perlman T, Shtabsky A, VandenBussche CJ, Ali SZ, Kolar Z, Pagni F, Bar D, Meiri E, Rosetta Genomics Group (2016). Analytical validity of a microRNA-based assay for diagnosing indeterminate thyroid FNA smears from routinely prepared cytology slides. Cancer Cytopathol.

[15] Nikiforov YE, Carty SE, Chiosea SI, Coyne C, Duvvuri U, Ferris RL, Gooding WE, Hodak SP, LeBeau SO, Ohori NP, Seethala RR, Tublin ME, Yip L, Nikiforova MN (2014). Highly accurate diagnosis of cancer in thyroid nodules with follicular neoplasm/suspicious for a follicular neoplasm cytology by ThyroSeq v2 next-generation sequencing assay. Cancer.

[16] Dennis G, Sherman BT, Hosack DA, Yang J, Gao W, Lane HC, Lempicki RA, Hosack DA, Yang J, Gao W, Lane HC, Lempicki RA (2003). DAVID: Database for Annotation, Visualization, and Integrated Discovery. Genome Biol.

[17] Borup R, Rossing M, Henao R, Yamamoto Y, Krogdahl A, Godballe C, Winther O, Kiss K, Christensen L, Høgdall E, Bennedbaek F, Nielsen FC (2010). Molecular signatures of thyroid follicular neoplasia. Endocr Relat Cancer.

[18] Finley DJ, Zhu B, Barden CB, Fahey TJ (2004). Discrimination of benign and malignant thyroid nodules by molecular profiling. Ann Surg.

[19] Weber F, Shen L, Aldred MA, Morrison CD, Frilling A, Saji M, Schuppert F, Broelsch CE, Ringel MD, Eng C (2005). Genetic classification of benign and malignant thyroid follicular neoplasia based on a three-gene combination. J Clin Endocrinol Metab.

[20] Giordano TJ, Au AY, Kuick R, Thomas DG, Rhodes DR, Wilhelm KG, Vinco M, Misek DE, Sanders D, Zhu Z, Ciampi R, Hanash S, Chinnaiyan A (2006). Delineation, functional validation, and bioinformatic evaluation of gene expression in thyroid follicular carcinomas with the PAX8-PPARG translocation. Clin Cancer Res.

[21] Fontaine JF, Mirebeau-Prunier D, Raharijaona M, Franc B, Triau S, Rodien P, Goëau-Brissonniére O, Karayan-Tapon L, Mello M, Houlgatte R, Malthiery Y, Savagner F (2009). Increasing the number of thyroid lesions classes in microarray analysis improves the relevance of diagnostic markers. PLoS One.

[22] Rossing M, Borup R, Henao R, Winther O, Vikesaa J, Niazi O, Godballe C, Krogdahl A, Glud M, Hjort-Sørensen C, Kiss K, Bennedbæk FN, Nielsen FC (2012). Down-regulation of microRNAs controlling tumourigenic factors in follicular thyroid carcinoma. J Mol Endocrinol.

[23] Stokowy T, Wojtaś B, Krajewska J, Stobiecka E, Dralle H, Musholt T, Hauptmann S, Lange D, Hegedüs L, Jarząb B, Krohn K, Paschke R, Eszlinger M (2015). A two miRNA classifier differentiates follicular thyroid carcinomas from follicular thyroid adenomas. Mol Cell Endocrinol.

[24] Reddi HV, Madde P, Milosevic D, Hackbarth JS, Algeciras-Schimnich A, McIver B, Grebe SK, Eberhardt NL (2011). The Putative PAX8/PPARγ Fusion Oncoprotein Exhibits Partial Tumor Suppressor Activity through Up-Regulation of Micro-RNA-122 and Dominant-Negative PPARγ Activity. Genes Cancer.

[25] Weber F, Teresi RE, Broelsch CE, Frilling A, Eng C (2006). A limited set of human MicroRNA is deregulated in follicular thyroid carcinoma. J Clin Endocrinol Metab.

[26] Mancikova V, Castelblanco E, Pineiro-Yanez E, Perales-Paton J, de Cubas AA, Inglada-Perez L, Matias-Guiu X, Capel I, Bella M, Lerma E, Riesco-Eizaguirre G, Santisteban P, Maravall F (2015). MicroRNA deep-sequencing reveals master regulators of follicular and papillary thyroid tumors. Mod Pathol.

[27] Stokowy T, Wojtaś B, Fujarewicz K, Jarząb B, Eszlinger M, Paschke R (2014). miRNAs with the potential to distinguish follicular thyroid carcinomas from benign follicular thyroid tumors: results of a meta-analysis. Horm Metab Res.

[28] Stokowy T, Eszlinger M, Świerniak M, Fujarewicz K, Jarząb B, Paschke R, Krohn K (2014). Analysis options for high-throughput sequencing in miRNA expression profiling. BMC Res Notes.

[29] Franc B, de la Salmonière P, Lange F, Hoang C, Louvel A, de Roquancourt A, Vildé F, Hejblum G, Chevret S, Chastang C (2003). Interobserver and intraobserver reproducibility in the histopathology of follicular thyroid carcinoma. Hum Pathol.

[30] Mazzaferri EL, Jhiang SM (1994). Long-term impact of initial surgical and medical therapy on papillary and follicular thyroid cancer. Am J Med.

[31] Letsas KP, Frangou-Lazaridis M, Skyrlas A, Tsatsoulis A, Malamou-Mitsi V (2005). Transcription factor-mediated proliferation and apoptosis in benign and malignant thyroid lesions. Pathol Int.

[32] Katoh R, Bray CE, Suzuki K, Komiyama A, Hemmi A, Kawaoi A, Oyama T, Sugai T, Sasou S (1995). Growth activity in hyperplastic and neoplastic human thyroid determined by an immunohistochemical staining procedure using monoclonal antibody MIB-1. Hum Pathol.

[33] Jang MH, Jung KC, Min HS (2015). The Diagnostic Usefulness of HMGA2, Survivin, CEACAM6, and SFN/14-3-3 δ in Follicular Thyroid Carcinoma. J Pathol Transl Med.

[34] Hirokawa M, Carney JA, Goellner JR, DeLellis RA, Heffess CS, Katoh R, Tsujimoto M, Kakudo K (2002). Observer variation of encapsulated follicular lesions of the thyroid gland. Am J Surg Pathol.

[35] Pfeifer A, Wojtas B, Oczko-Wojciechowska M, Kukulska A, Czarniecka A, Eszlinger M, Musholt T, Stokowy T, Swierniak M, Stobiecka E, Rusinek D, Tyszkiewicz T, Kowal M (2013). Molecular differential diagnosis of follicular thyroid carcinoma and adenoma based on gene expression profiling by using formalin-fixed paraffin-embedded tissues. BMC Med Genomics.

[36] Fryknäs M, Wickenberg-Bolin U, Göransson H, Gustafsson MG, Foukakis T, Lee JJ, Landegren U, Höög A, Larsson C, Grimelius L, Wallin G, Pettersson U, Isaksson A (2006). Molecular markers for discrimination of benign and malignant follicular thyroid tumors. Tumour Biol.

[37] Hébrant A, Dom G, Dewaele M, Andry G, Trésallet C, Leteurtre E, Dumont JE, Maenhaut C (2012). mRNA expression in papillary and anaplastic thyroid carcinoma: molecular anatomy of a killing switch. PLoS One.

[38] Schmid KW, Farid NR (2006). How to define follicular thyroid carcinoma?. Virchows Arch.

[39] Derwahl M, Studer H (2002). Hyperplasia versus adenoma in endocrine tissues: are they different?. Trends Endocrinol Metab.

[40] Hébrant A, Floor S, Saiselet M, Antoniou A, Desbuleux A, Snyers B, La C, de Saint Aubain N, Leteurtre E, Andry G, Maenhaut C (2014). miRNA expression in anaplastic thyroid carcinomas. PLoS One.

[41] Belge G, Meyer A, Klemke M, Burchardt K, Stern C, Wosniok W, Loeschke S, Bullerdiek J (2008). Upregulation of HMGA2 in thyroid carcinomas: a novel molecular marker to distinguish between benign and malignant follicular neoplasias. Genes Chromosomes Cancer.

[42] Lappinga PJ, Kip NS, Jin L, Lloyd RV, Henry MR, Zhang J, Nassar A (2010). HMGA2 gene expression analysis performed on cytologic smears to distinguish benign from malignant thyroid nodules. Cancer Cytopathol.

[43] Prasad NB, Kowalski J, Tsai HL, Talbot K, Somervell H, Kouniavsky G, Wang Y, Dackiw AP, Westra WH, Clark DP, Libutti SK, Umbricht CB, Zeiger MA (2012). Three-gene molecular diagnostic model for thyroid cancer. Thyroid.

[44] Jin L, Lloyd RV, Nassar A, Lappinga PJ, Sebo TJ, Swartz K, Seys AR, Erickson-Johnson MR, Roth CW, Evers BR, Oliveira AM, Zhang J (2011). HMGA2 expression analysis in cytological and paraffin-embedded tissue specimens of thyroid tumors by relative quantitative RT-PCR. Diagn Mol Pathol.

[45] Rogalla P, Drechsler K, Kazmierczak B, Rippe V, Bonk U, Bullerdiek J (1997). Expression of HMGI-C, a member of the high mobility group protein family, in a subset of breast cancers: relationship to histologic grade. Mol Carcinog.

[46] Mahajan A, Liu Z, Gellert L, Zou X, Yang G, Lee P, Yang X, Wei JJ (2010). HMGA2: a biomarker significantly overexpressed in high-grade ovarian serous carcinoma. Mod Pathol.

[47] Pallante P, Sepe R, Puca F, Fusco A (2015). High mobility group a proteins as tumor markers. Front Med (Lausanne).

[48] Meyer B, Loeschke S, Schultze A, Weigel T, Sandkamp M, Goldmann T, Vollmer E, Bullerdiek J (2007). HMGA2 overexpression in non-small cell lung cancer. Mol Carcinog.

[49] Tomás G, Tarabichi M, Gacquer D, Hébrant A, Dom G, Dumont JE, Keutgen X, Fahey TJ, Maenhaut C, Detours V (2012). A general method to derive robust organ-specific gene expression-based differentiation indices: application to thyroid cancer diagnostic. Oncogene.

[50] Finn SP, Smyth P, Cahill S, Streck C, O'Regan EM, Flavin R, Sherlock J, Howells D, Henfrey R, Cullen M, Toner M, Timon C, O'Leary JJ, Sheils OM (2007). Expression microarray analysis of papillary thyroid carcinoma and benign thyroid tissue: emphasis on the follicular variant and potential markers of malignancy. Virchows Arch.

[51] Furuhashi M, Saitoh S, Shimamoto K, Miura T (2015). Fatty Acid-Binding Protein 4 (FABP4): Pathophysiological Insights and Potent Clinical Biomarker of Metabolic and Cardiovascular Diseases. Clin Med Insights Cardiol.

[52] Dobson ME, Diallo-Krou E, Grachtchouk V, Yu J, Colby LA, Wilkinson JE, Giordano TJ, Koenig RJ (2011). Pioglitazone induces a proadipogenic antitumor response in mice with PAX8-PPARgamma fusion protein thyroid carcinoma. Endocrinology.

[53] Gorbenko O, Panayotou G, Zhyvoloup A, Volkova D, Gout I, Filonenko V (2010). Identification of novel PTEN-binding partners: PTEN interaction with fatty acid binding protein FABP4. Mol Cell Biochem.

[54] Ban Y, Yamamoto G, Takada M, Hayashi S, Ban Y, Shimizu K, Akasu H, Igarashi T, Bando Y, Tachikawa T, Hirano T (2012). Proteomic profiling of thyroid papillary carcinoma. J Thyroid Res.

[55] Schulze A, Harris AL (2012). How cancer metabolism is tuned for proliferation and vulnerable to disruption. Nature.

[56] Currie E, Schulze A, Zechner R, Walther TC, Farese RV (2013). Cellular fatty acid metabolism and cancer. Cell Metab.

[57] Guo S, Qiu L, Wang Y, Qin X, Liu H, He M, Zhang Y, Li Z, Chen X (2014). Tissue imaging and serum lipidomic profiling for screening potential biomarkers of thyroid tumors by matrix-assisted laser desorption/ionization-Fourier transform ion cyclotron resonance mass spectrometry. Anal Bioanal Chem.

[58] Wojakowska A, Chekan M, Marczak Ł, Polanski K, Lange D, Pietrowska M, Widlak P (2015). Detection of metabolites discriminating subtypes of thyroid cancer: molecular profiling of FFPE samples using the GC/MS approach. Mol Cell Endocrinol.

[59] von Roemeling CA, Marlow LA, Pinkerton AB, Crist A, Miller J, Tun HW, Smallridge RC, Copland JA (2015). Aberrant lipid metabolism in anaplastic thyroid carcinoma reveals stearoyl CoA desaturase 1 as a novel therapeutic target. J Clin Endocrinol Metab.

[60] Riesco-Eizaguirre G, Santisteban P (2007). New insights in thyroid follicular cell biology and its impact in thyroid cancer therapy. Endocr Relat Cancer.

[61] Robbins HL, Hague A (2016). The PI3K/Akt Pathway in Tumors of Endocrine Tissues. Front Endocrinol (Lausanne).

[62] Matsuda S, Nakanishi A, Wada Y, Kitagishi Y (2013). Roles of PI3K/AKT/PTEN Pathway as a Target for Pharmaceutical Therapy. Open Med Chem J.

[63] Saiselet M, Pita JM, Augenlicht A, Dom G, Tarabichi M, Fimereli D, Dumont JE, Detours V, Maenhaut C (2016). miRNA expression and function in thyroid carcinomas: a comparative and critical analysis and a model for other cancers. Oncotarget.

[64] Floor SL, Hebrant A, Pita JM, Saiselet M, Trésallet C, Libert F, Andry G, Dumont JE, van Staveren WC, Maenhaut C (2014). MiRNA expression may account for chronic but not for acute regulation of mRNA expression in human thyroid tumor models. PLoS One.

[65] Dal Maso L, Bosetti C, La Vecchia C, Franceschi S (2009). Risk factors for thyroid cancer: an epidemiological review focused on nutritional factors. Cancer Causes Control.

[66] Yoon JH, Kim EK, Youk JH, Moon HJ, Kwak JY (2014). Better understanding in the differentiation of thyroid follicular adenoma, follicular carcinoma, and follicular variant of papillary carcinoma: a retrospective study. Int J Endocrinol.

[67] Ghossein R (2009). Problems and controversies in the histopathology of thyroid carcinomas of follicular cell origin. Arch Pathol Lab Med.

[68] Cancer T, Atlas G, Cancer Genome Atlas Research Network (2014). Integrated genomic characterization of papillary thyroid carcinoma. Cell.

[69] Mehrzad R, Nishino M, Connolly J, Wang H, Mowschenson P, Hasselgren PO (2016). The relationship between the follicular variant of papillary thyroid cancer and follicular adenomas. Surgery.

[70] Nikiforov YE, Seethala RR, Tallini G, Baloch ZW, Basolo F, Thompson LD, Barletta JA, Wenig BM, Al Ghuzlan A, Kakudo K, Giordano TJ, Alves VA, Khanafshar E (2016). Nomenclature Revision for Encapsulated Follicular Variant of Papillary Thyroid Carcinoma: A Paradigm Shift to Reduce Overtreatment of Indolent Tumors. JAMA Oncol.

[71] Stratton MR, Campbell PJ, Futreal PA (2009). The cancer genome. Nature.

[72] Xing M (2013). Molecular pathogenesis and mechanisms of thyroid cancer. Nat Rev Cancer.

[73] Kondo T, Ezzat S, Asa SL (2006). Pathogenetic mechanisms in thyroid follicular-cell neoplasia. Nat Rev Cancer.

[74] Saiselet M, Floor S, Tarabichi M, Dom G, Hébrant A, van Staveren WC, Maenhaut C (2012). Thyroid cancer cell lines: an overview. Front Endocrinol (Lausanne).

[75] Hébrant A, Van Sande J, Roger PP, Patey M, Klein M, Bournaud C, Savagner F, Leclère J, Dumont JE, van Staveren WC, Maenhaut C (2009). Thyroid gene expression in familial nonautoimmune hyperthyroidism shows common characteristics with hyperfunctioning autonomous adenomas. J Clin Endocrinol Metab.

[76] Delys L, Detours V, Franc B, Thomas G, Bogdanova T, Tronko M, Libert F, Dumont JE, Maenhaut C (2007). Gene expression and the biological phenotype of papillary thyroid carcinomas. Oncogene.

[77] Simon R, Lam A, Li MC, Ngan M, Menenzes S, Zhao Y (2007). Analysis of gene expression data using BRB-ArrayTools. Cancer Inform.

[78] Tusher VG, Tibshirani R, Chu G (2001). Significance analysis of microarrays applied to the ionizing radiation response. Proc Natl Acad Sci USA.

[79] Gentleman RC, Carey VJ, Bates DM, Bolstad B, Dettling M, Dudoit S, Ellis B, Gautier L, Ge Y, Gentry J, Hornik K, Hothorn T, Huber W (2004). Bioconductor: open software development for computational biology and bioinformatics. Genome Biol.

[80] Reich M, Liefeld T, Gould J, Lerner J, Tamayo P, Mesirov JP, Chapman SJ, Khor CC, Davies WH, Hedley EL, Segal S, Moore CE, Knox K (2006). GenePattern 2.0. Nat Genet.

[81] Subramanian A, Tamayo P, Mootha VK, Mukherjee S, Ebert BL, Gillette MA, Paulovich A, Pomeroy SL, Golub TR, Lander ES, Mesirov JP (2005). Gene set enrichment analysis: a knowledge-based approach for interpreting genome-wide expression profiles. Proc Natl Acad Sci USA.

[82] Mi H, Poudel S, Muruganujan A, Casagrande JT, Thomas PD (2016). PANTHER version 10: expanded protein families and functions, and analysis tools. Nucleic Acids Res.

