# Supplementary material for: Thyroid follicular adenomas and carcinomas: molecular profiling provides evidence for a continuous evolution
Source: Oncotarget. 2017 Dec 8;9(12):10343–59. doi: 10.18632/oncotarget.23130 (PMC5828225; doi:10.18632/oncotarget.23130)
Supplement: Supplementary file 1 [file oncotarget-09-10343-s001.pdf]

# Thyroid follicular adenomas and carcinomas: molecular profiling provides evidence for a continuous evolution

## SUPPLEMENTARY MATERIALS

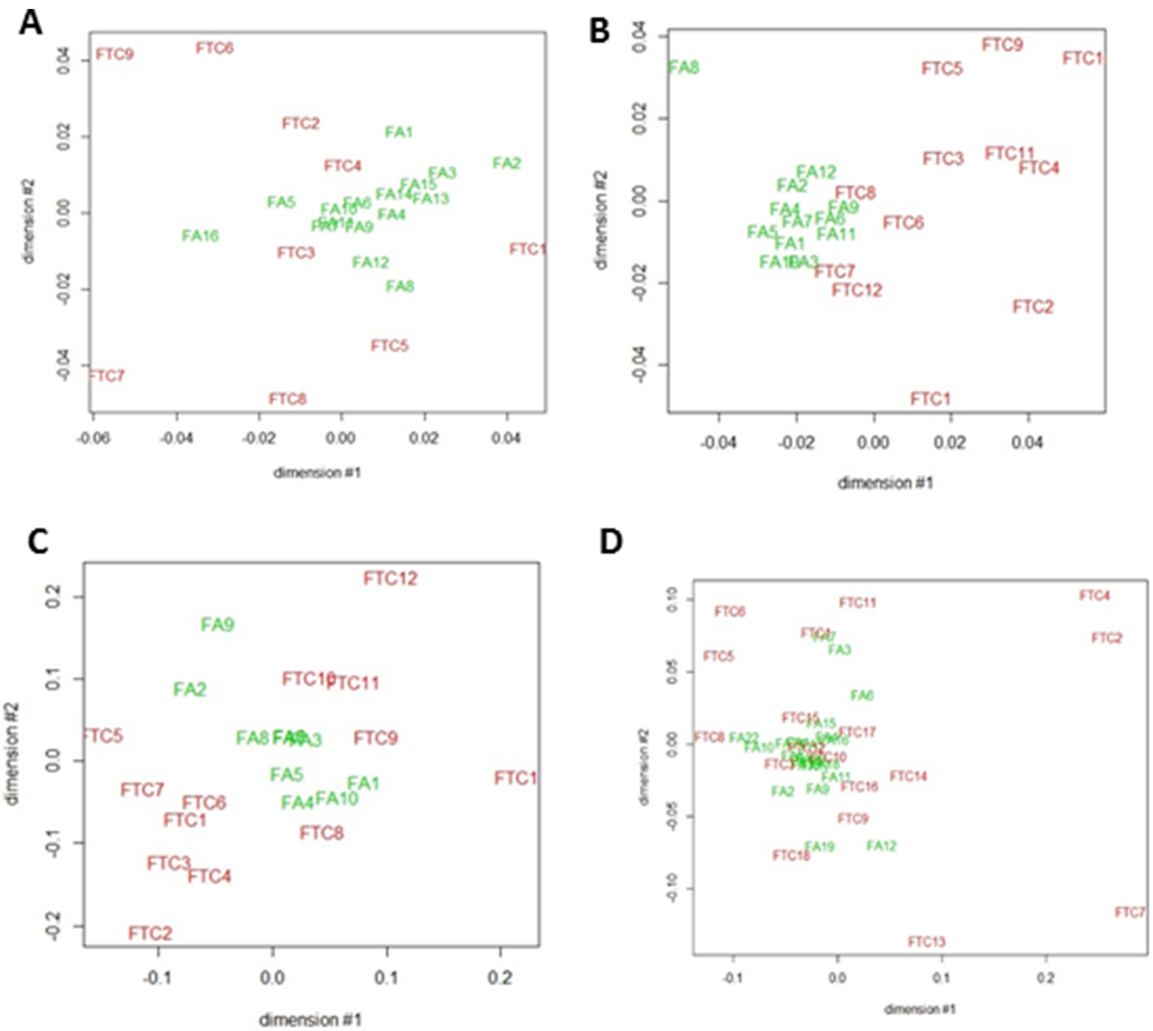

**Supplementary Figure 1:** Multidimensional Scaling of microarray mRNA expression data from published FA and FTC datasets ((**A**) Finley et al. [18], (**B**) Weber et al. [19], (**C**) Giordano et al. [20], (**D**) Borup et al [17]).

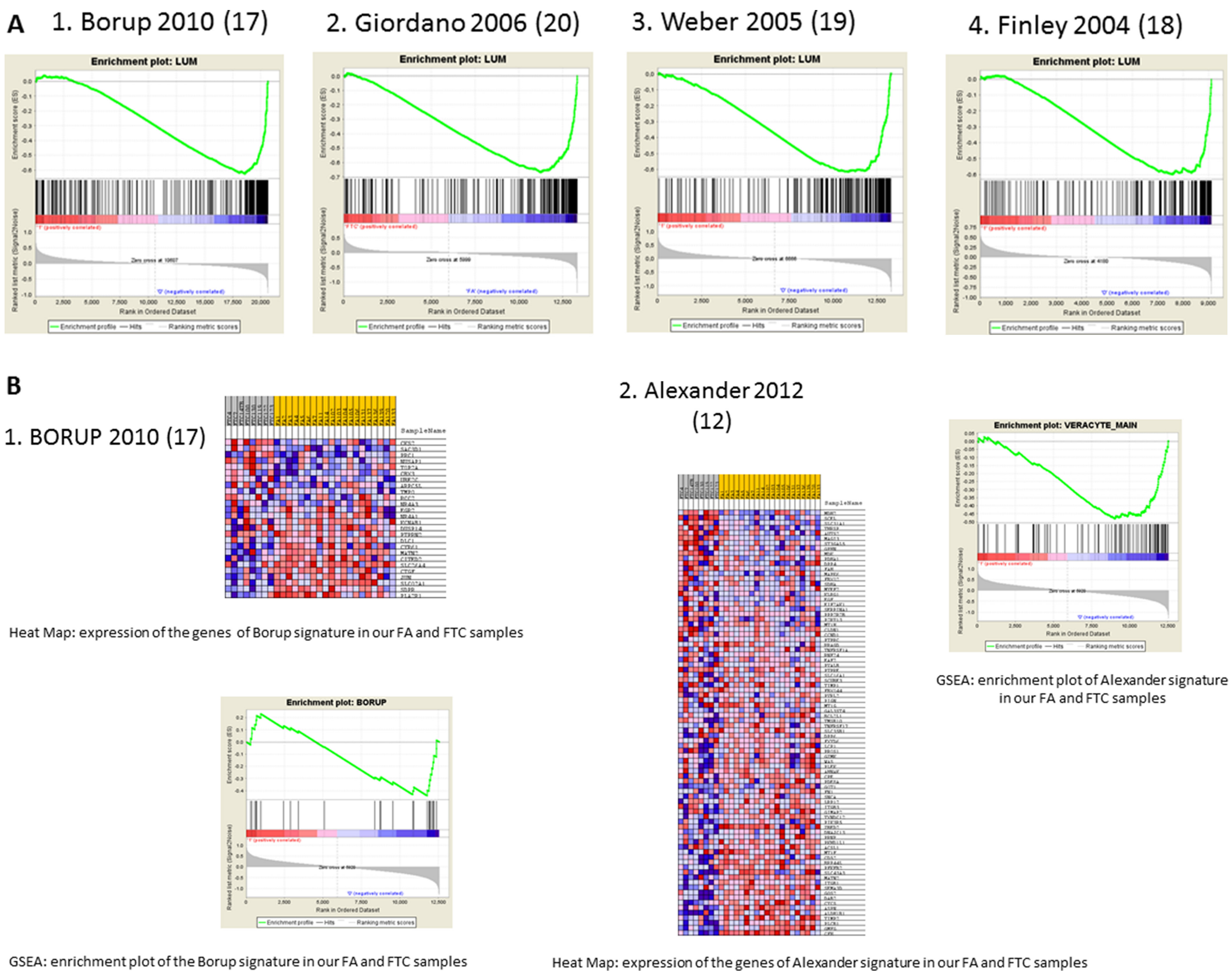


**Supplementary Figure 2: Results of GSEA (Gene Set Enrichment Analysis).** (**A**) Enrichment of our differentially expressed genes obtained with SAM (regulated more than 2 times) in published FA and FTC datasets [17–20]. (**B**) Enrichment of published mRNA expression signatures [12, 17] in our microarray dataset.

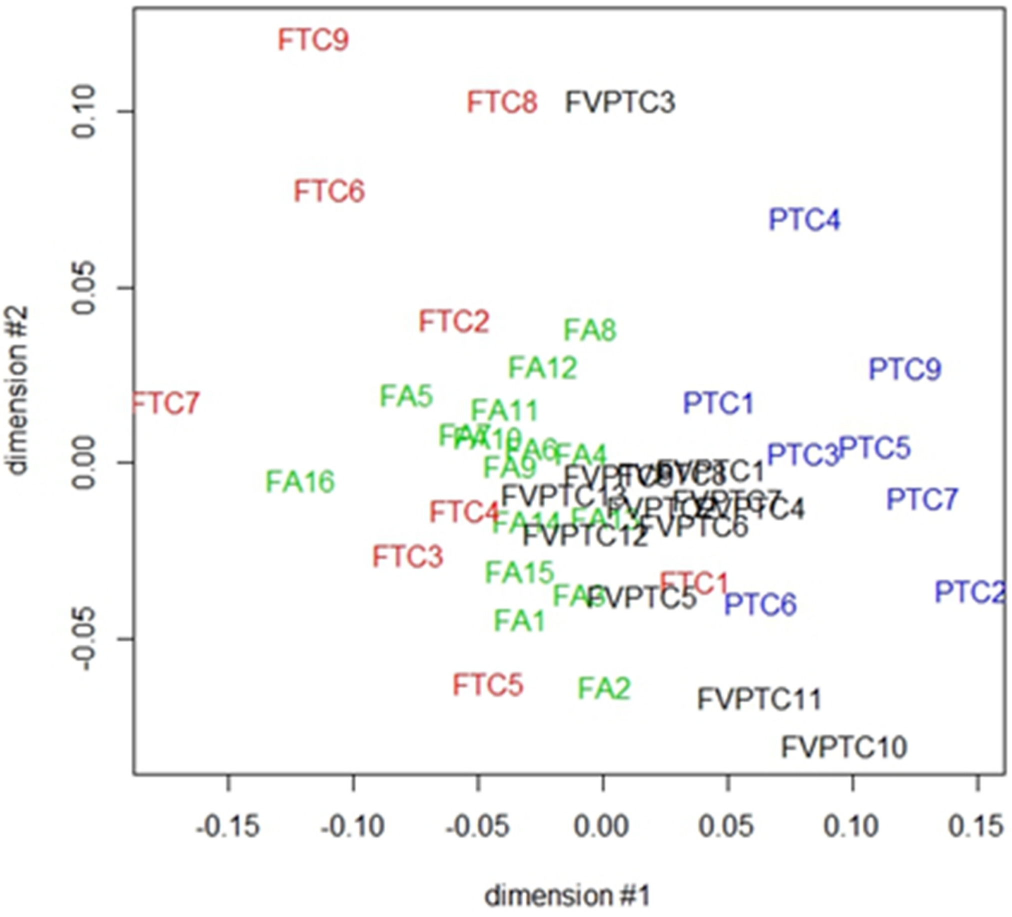


**Supplementary Figure 3: Multidimensional Scaling of the mRNA expression data for FA, FTC, FVPTC and PTC from Finley's group [18].**

**Supplementary Table 1: Patient clinical information for the FA and FTC samples used for microarray analyses.** See_Supplementary_Table 1

**Supplementary Table 2: List of the 294 genes significantly regulated between FA and FTC.** See_ Supplementary_Table 2

**Supplementary Table 3: Classification of independent FA (*n* = 8) and FTC (*n* = 11) with the qRT-PCR expression data for HMGA2, CRABP1, and FABP4, using the KNNX validation algorithm**

| | | Predicted | |
|---|---|---|---|
| | | FA | FTC |
| True | FA (8) | 88% (7) | 12% (1) |
| | FTC (11) | 9% (1) | 91% (10) |

Confusion matrix: 91% of FTC and 88% of FA were well classified.

**Supplementary Table 4: List of deregulated miRNAs between FA and FTC (by compiling our data and those of the literature (red: upregulated in FTC, green: downregulated in FTC) in literature ((24)(25)(26)(23)(22)(27)(28)) and house data.** See_Supplementary_Table 4

**Supplementary Table 5: David database pathway analysis with the mRNAs that are targets of our regulated miRs (obtained by miRDB tool), and regulated in our microarray data: significantly regulated gene ontology biological processes**

| Category | Term | Fold Enrichment | Bonferroni | Benjamini | FDR |
|---|---|---|---|---|---|
| GOTERM_BP_FAT | GO:0045859~regulation of protein kinase activity | 3.92115942 | | | 3.03230816 |
| GOTERM_BP_FAT | GO:0043549~regulation of kinase activity | 3.789355742 | | | 3.73771274 |
| GOTERM_BP_FAT | GO:0010033~response to organic substance | 2.710186469 | | | 4.07344125 |
| GOTERM_BP_FAT | GO:0051338~regulation of transferase activity | 3.63655914 | | | 4.78837997 |

**Supplementary Table 6: Sequences of the primers used for qRT-PCR (fwd: forward, rev: reverse)**

| |
|---|
| FABP4-fwd : 5′-GCC-AGG-AAT-TTG-ACG-AAG-TCA-C -3′ |
| FABP4-rev: 5′-TTC-TGC-ACA-TGT-ACC-AGG-ACA-C -3′ |
| CRABP-fwd: 5′ TGT-GAA-CGC-CAT-GCT-GAG 3′ |
| CRABP1-rev: 5′ CTC-ACG-GGT-CCA-GTA-GGT-TT 3′ |
| HMGA2-fwd:5′-AGT-CCC-TCT-AAA-GCA-CAA-AAG-3′ |
| HMGA2-rev: 5′-CCA-TTT-CCT-AGG-TCT-GCC-TC-3′ |
| DCN-fwd: 5′-TGG-CAA-CAA-AAT-CAG-CAG-AG-3′ |
| DCN-rev: 5′-CCC-ATT-GTC-AAC-AGC-AGA-GA-3′ |
| TGFBR2-fwd: 5′‘-CTG-GTG-CTC-TGG-GAA-ATG-AC-3′ |
| TGFBR2-rev: 5′-CAA-CAC-GTT-GTC-CTT-CAT-GC-3′ |
| PLAG1-fwd: 5′-CAC-AGG-AGA-GAG-GCC-CTA-CA-3′ |
| PLAG1-rev: 5′-GGT-CGT-GTG-TAT-GGA-GGT-GA-3′ |
| GDF15-fwd: 5′-CTC-CAG-ATT-CCG-AGA-GTT-GC-3′ |
| GDF15-rev: 5′-AGA-GAT-ACG-CAG-GTG-CAG-GT-3′ |
| ITGA1-fwd: 5′-ACA-GCG-AAG-AAC-CTC-CTG-AA-3′ |
| ITGA1-rev: 5′-GAC-TGT-CTC-ATT-GGC-AGC-AA-3′ |
| SCEL-fwd: 5′-CCC-AAG-GAT-GGA-TAT-CAG-GA-3′ |
| SCEL-rev: 5′-TCT-ACA-CCC-AAG-GGT-TTT-CG-3′ |
| SGNE1-fwd: 5′-GGT-ACC-CAG-ACC-CTC-CAA-AT-3′ |
| SGNE1-rev: 5′-TCG-TCT-CTC-TCC-TCC-CTT-CA-3′ |
| LUM-fwd: 5′-CCT-GGT-TGA-GCT-GGA-TCT-GT-3′ |
| LUM-rev: 5′-TAG-GAT-AAT-GGC-CCC-AGG-A-3′ |
